# Supplementary material for: Triplet correlations in Cooper pair splitters realized in a two-dimensional electron gas
Source: Nat Commun. 2023 Aug 12;14:4876. doi: 10.1038/s41467-023-40551-z (PMC10423214; doi:10.1038/s41467-023-40551-z)
Supplement: Supplementary file 1 — Supplementary Information [file 41467_2023_40551_MOESM1_ESM.pdf]

Triplet correlations in Cooper pair splitters realized in a two-dimensional electron gas

Qingzhen Wang,[1,†] Sebastiaan L.D. ten Haaf,[1,†] Ivan Kulesh,[1,†] Di Xiao,[2]
Candice Thomas,[2] Michael J. Manfra,[2,3,4,5] and Srijit Goswami[1,*]

[1]*QuTech and Kavli Institute of Nanoscience, Delft University of Technology, Delft, 2600 GA, The Netherlands*
[2]*Department of Physics and Astronomy, Purdue University, West Lafayette, 47907, Indiana, USA*
[3]*Elmore School of Electrical and Computer Engineering,
Purdue University, West Lafayette, 47907, Indiana, USA*
[4]*School of Materials Engineering, Purdue University, West Lafayette, 47907, Indiana, USA*
[5]*Microsoft Quantum Lab, West Lafayette, 47907, Indiana, USA*

† These authors contributed equally to this work.

* s.goswami@tudelft.nl

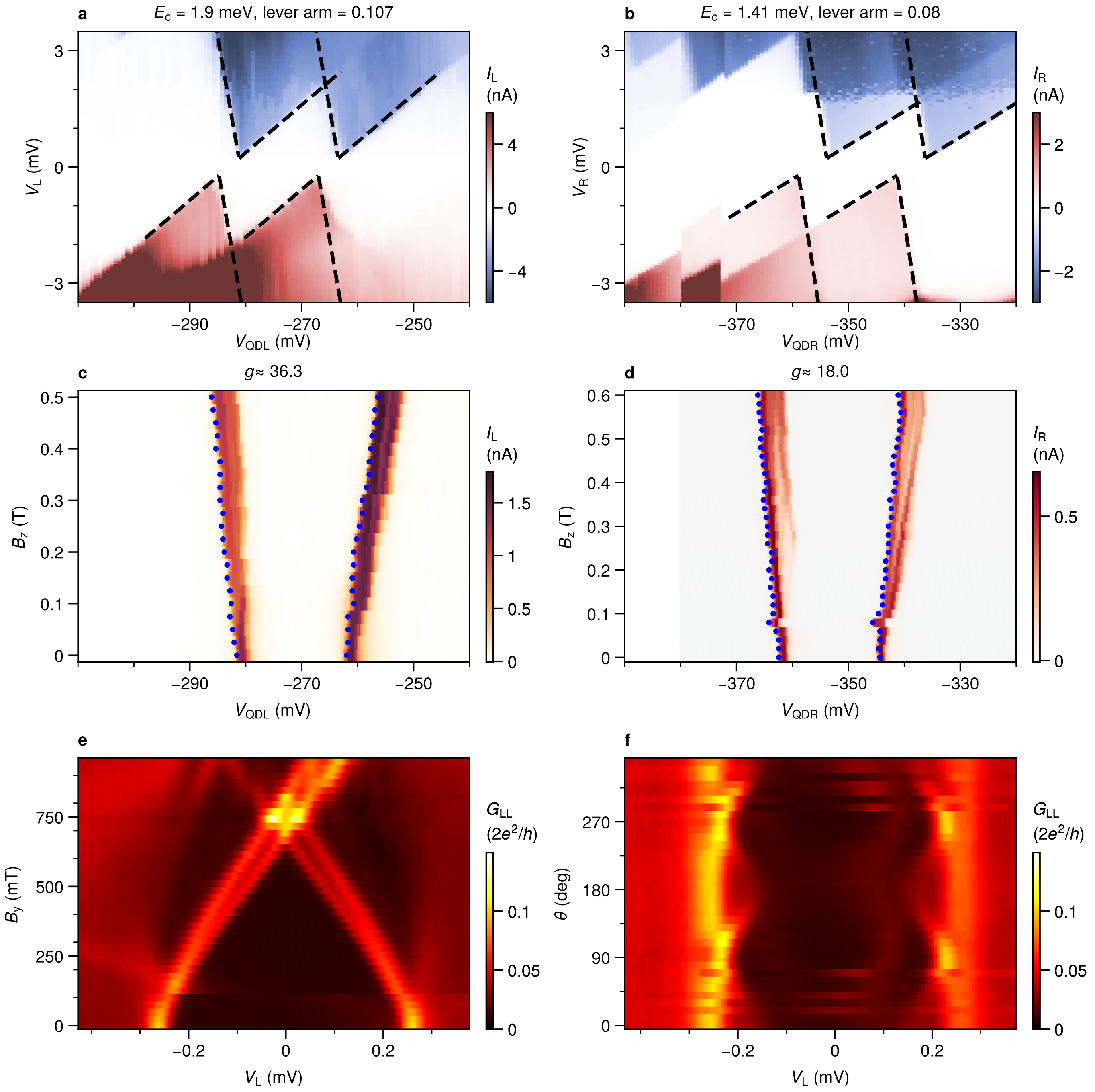

FIG. S1. **Quantum dot and ABS characterization.** Extraction of charging energy E_c and lever arm for (**a**) QDL and (**b**) QDR from Coulomb diamonds shown in Fig 1 of the main text. Evolution of Coulomb peaks with B_z for (**c**) QDL and (**d**) QDR shows Zeeman splitting causing the separation between the resonances to increase. Bias voltages are $(V_L, V_R) = (-450\,\mu V, 0)$ for the left measurement, and $(0, -350\,\mu V)$ for the right measurement. g-factors of -36 and -18 are derived for the QDL and QDR respectively. **(e)** Measured local conductance G_{LL} of the hybrid section with increasing magnetic field $B \parallel B_y$ at $V_{ABS} = -245\,$mV. **(f)** Field rotation in the y-z plane with a fiedld magnitude of 100 mT, showing a slight anisotropy of the ABS energy.

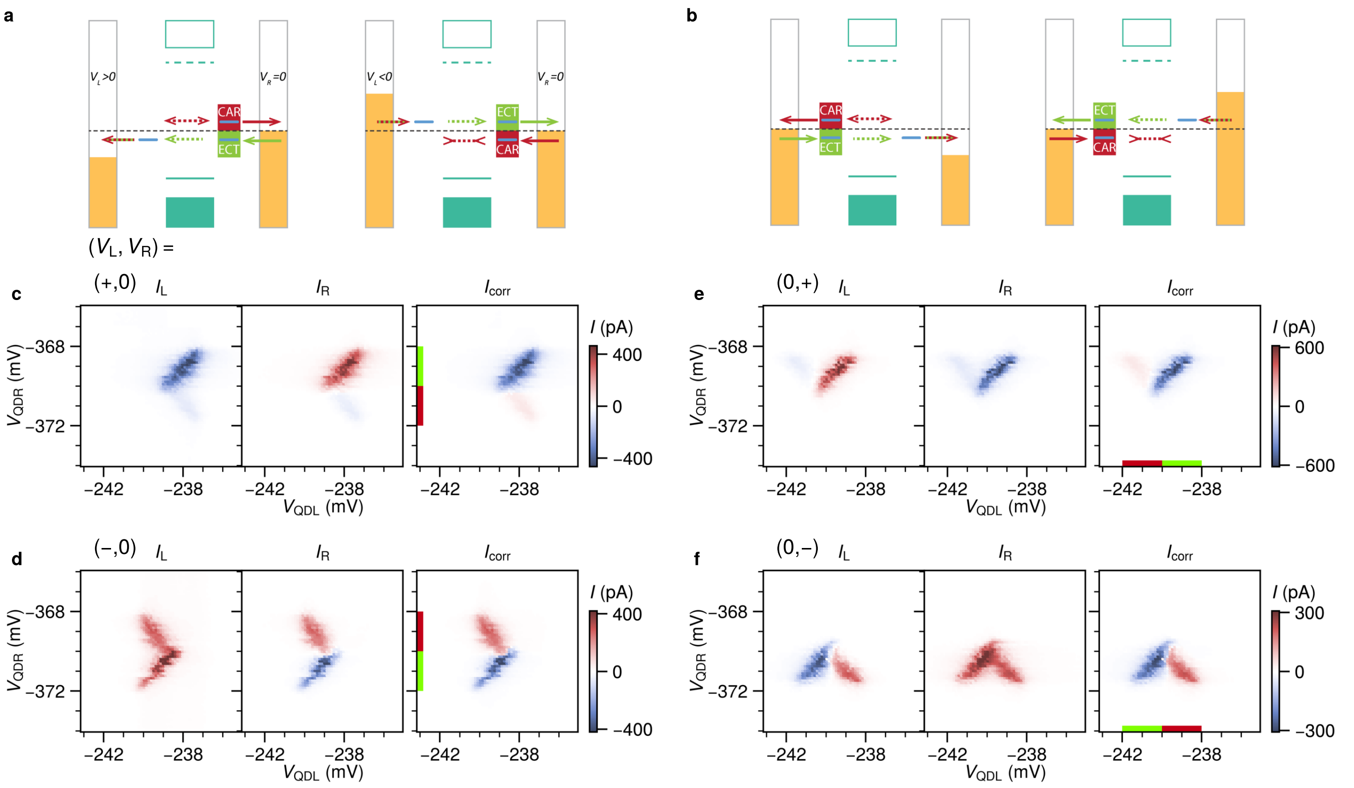

FIG. S2. **Simultaneous measurement of ECT and CAR**. Energy diagrams for the expected transport when biasing only the **(a)** left or **(b)** right side of the device. **(c)-(f)** Corresponding measurements with the bias polarities labelled in the top left corners. Two distinct features with opposite slope appear in each charge stability diagram. This is because both ECT and CAR can now occur, depending on the position of the QD levels with respect to the grounded side. Red (CAR) and green (ECT) bars indicate the corresponding gate voltage ranges in which each process is allowed.

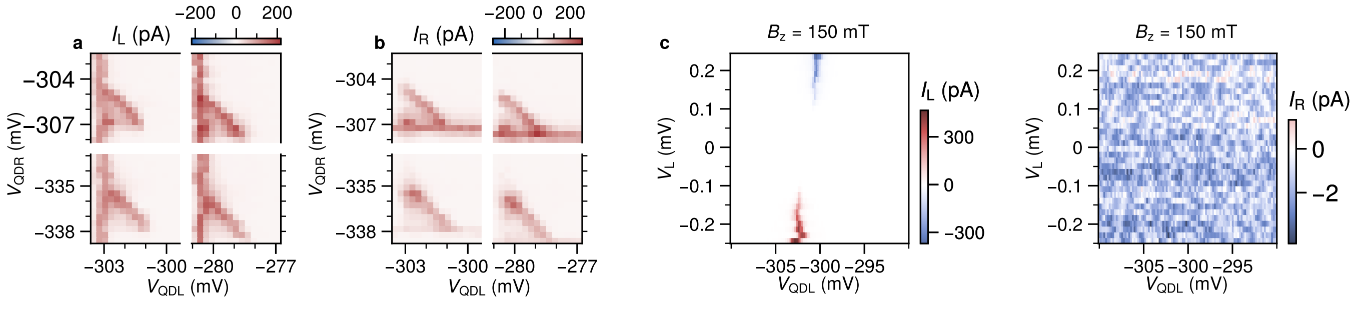

FIG. S3. **Filtering local transport processes.** A key requirement for isolating CAR and ECT is the ability to filter out local processes. For the measurements presented in the main text it was ensured that the applied biases remained below the energy of any subgap states. With applied biases of -120 μV on both sides and an external magnetic field $B_z = 150$ mT, additional currents appear in **(a)** I_L and **(b)** I_R on top of the currents arising through CAR. Fixing V_{QDR} at -320 mV (i.e., putting the right dot off-resonance), I_L and I_R are measured as a function of the left bias V_L and left plunger gate V_{QDL}. (**c**) Current arising through local processes appears once the applied bias exceeds the ABS energy. No current is detected in I_R.

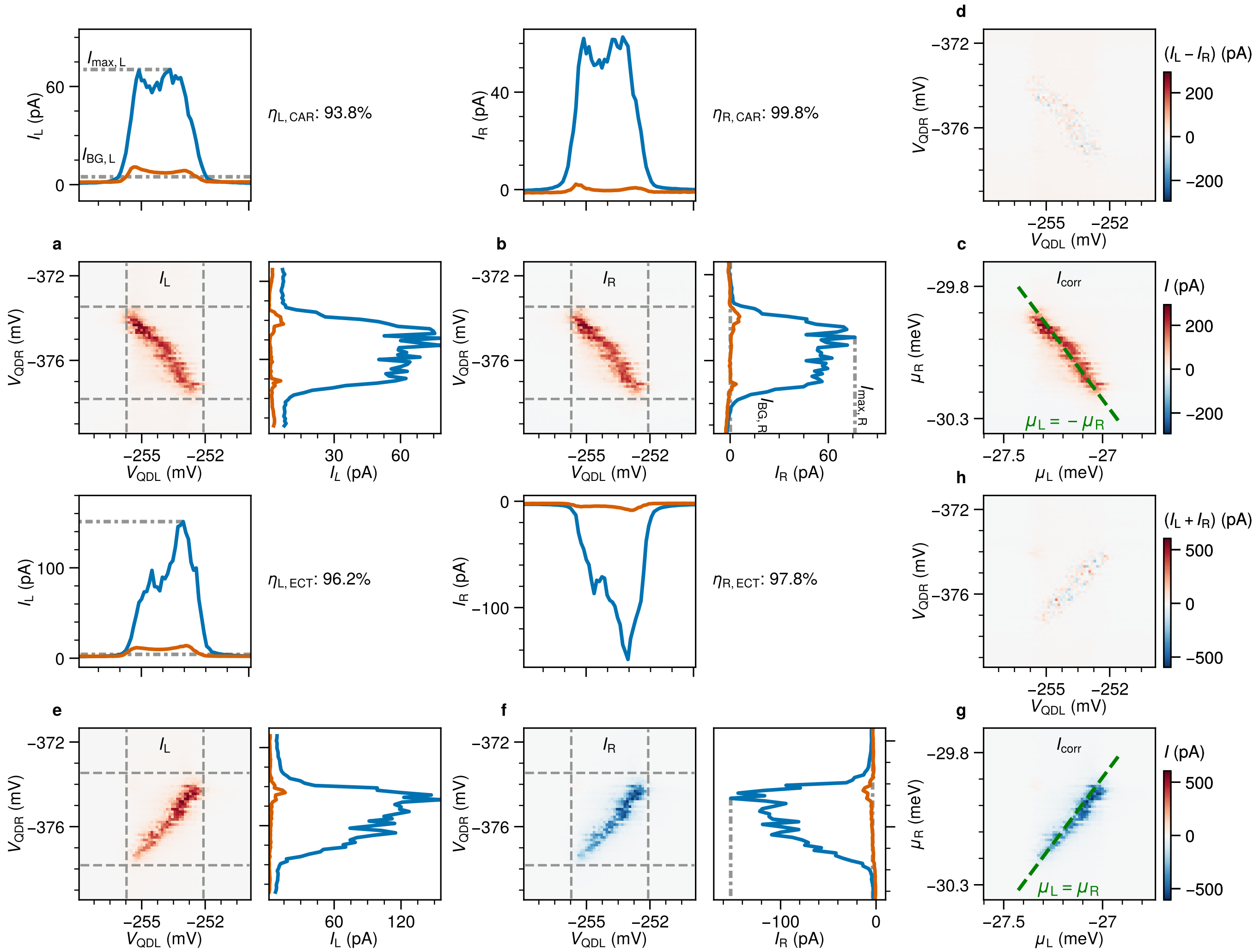

FIG. S4. **Cooper pair splitting efficiency.** Analysis of the data presented in Fig. 2. **(a,b)** Measured currents I_{L} and I_{R} arising from CAR. The top panel shows I_{L} averaged over the V_{QDR} range indicated by the horizontal grey lines (blue) and the background trace averaged outside the horizontal grey lines (orange). The right panel shows I_{L} averaged over the V_{QDL} range indicated by the vertical grey lines and the background trace averaged outside the vertical grey lines. The efficiency of each junction is defined as $\eta_j = 1 - \frac{I_{BG,j}}{I_{max,j}}$ where the $I_{BG,j}$ is the average value of the background trace and $I_{max,j}$ is the maximum of the averaged I_j. We extract an η of about 94% for the left junction and 99% for the right junction. Taking the product this gives a combined efficiency of $\eta_L \eta_R$ of about 93%. Using the lever arms extracted from the Coulomb diamonds (Fig. S1), the correlated current I_{corr} is plotted as a function of μ_{L} and μ_{R} in **(c)**. The dashed line corresponds to $\mu_{\mathrm{L}} = -\mu_{\mathrm{R}}$, confirming that transport occurs when the dot levels are anti-aligned. Calculating $I_{\mathrm{L}} - I_{\mathrm{R}}$ shows very small remaining current, verifying $I_{\mathrm{L}} = I_{\mathrm{R}}$ **(d)**. **(e,f)** Similar analysis of I_{L}, I_{R} and averaged currents for ECT. The combined efficiency is again found to be about 93%. **(g)** I_{corr} in μ_{L}- μ_{R} space, together with the line where $\mu_{\mathrm{L}} = \mu_{\mathrm{R}}$, showing that transport takes place when the QD levels are aligned. **(h)** Plotting $I_{\mathrm{L}} + I_{\mathrm{R}}$ shows again that little signal remains, highlighting that $I_{\mathrm{L}} = -I_{\mathrm{R}}$ for ECT.

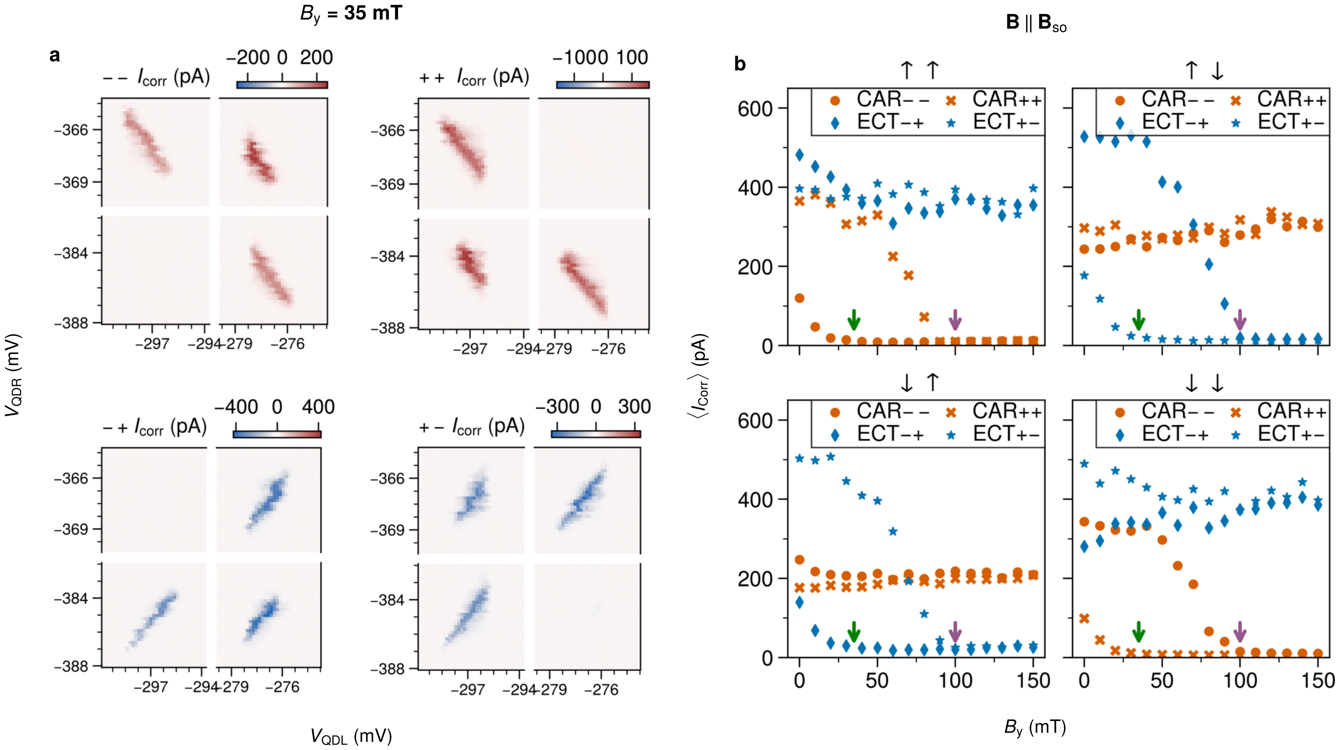

FIG. S5. **Field evolution of ECT and CAR. (a)** The charge stability diagrams at $B_y = 35$ mT, of the same resonances used in the main text, show that the weakened transitions in each bias configuration (circled in Fig. 2c) are now completely blocked. This is interpreted as a result of the magnetic field overcoming the hyperfine interaction in the QDs. **(b)** Spin-filtered measurements of $\langle I_{corr} \rangle$ for ECT and CAR as a function of magnetic field $B_y \parallel \mathbf{B}_{SO}$, for each bias configuration. Two transitions are present in each quadrant. Above roughly 35 mT (green arrows) the Pauli-blockaded process in each quadrant becomes fully suppressed. Above 100 mT (purple arrows) the Zeeman splitting exceeds the applied biases ($|100\ \mu V|$) such that only the spin-preserving processes remain (i.e. opposite-spin CAR and equal-spin ECT).

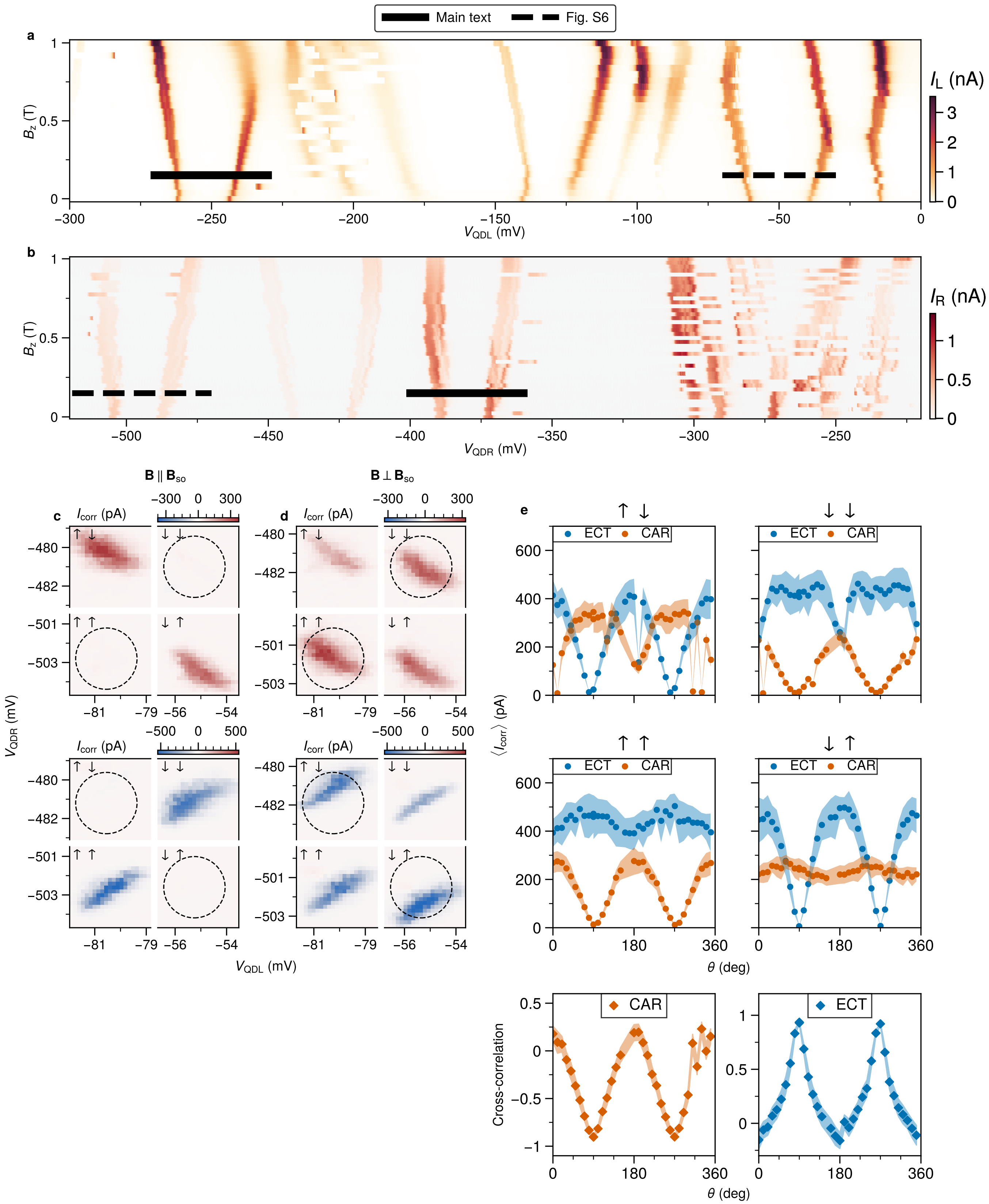

FIG. S6. **Field angle dependence with another pair of QD resonances.** **(a,b)** Field evolution of Coulomb resonances in the QDs over an extended range of V_{QDL} and V_{QDR}. The effect of Zeeman splitting is observed for multiple orbitals in each QD. Solid black lines indicate the orbitals used in the main text, while the dashed lines indicate different orbitals used for data presented here. **(c,d)** Repetition of measurements presented in Fig. 4(c,d) for the second pair of resonances. Unconventional processes (equal-spin CAR and opposite-spin ECT) are fully suppressed at $B \parallel B_{\mathrm{SO}}$ and recovered at $B \perp B_{\mathrm{SO}}$. A full field rotation **(e)** yields similar behaviours of $\langle I_{\mathrm{corr}} \rangle$ and cross-correlation to the dependence shown in Fig. 4f.

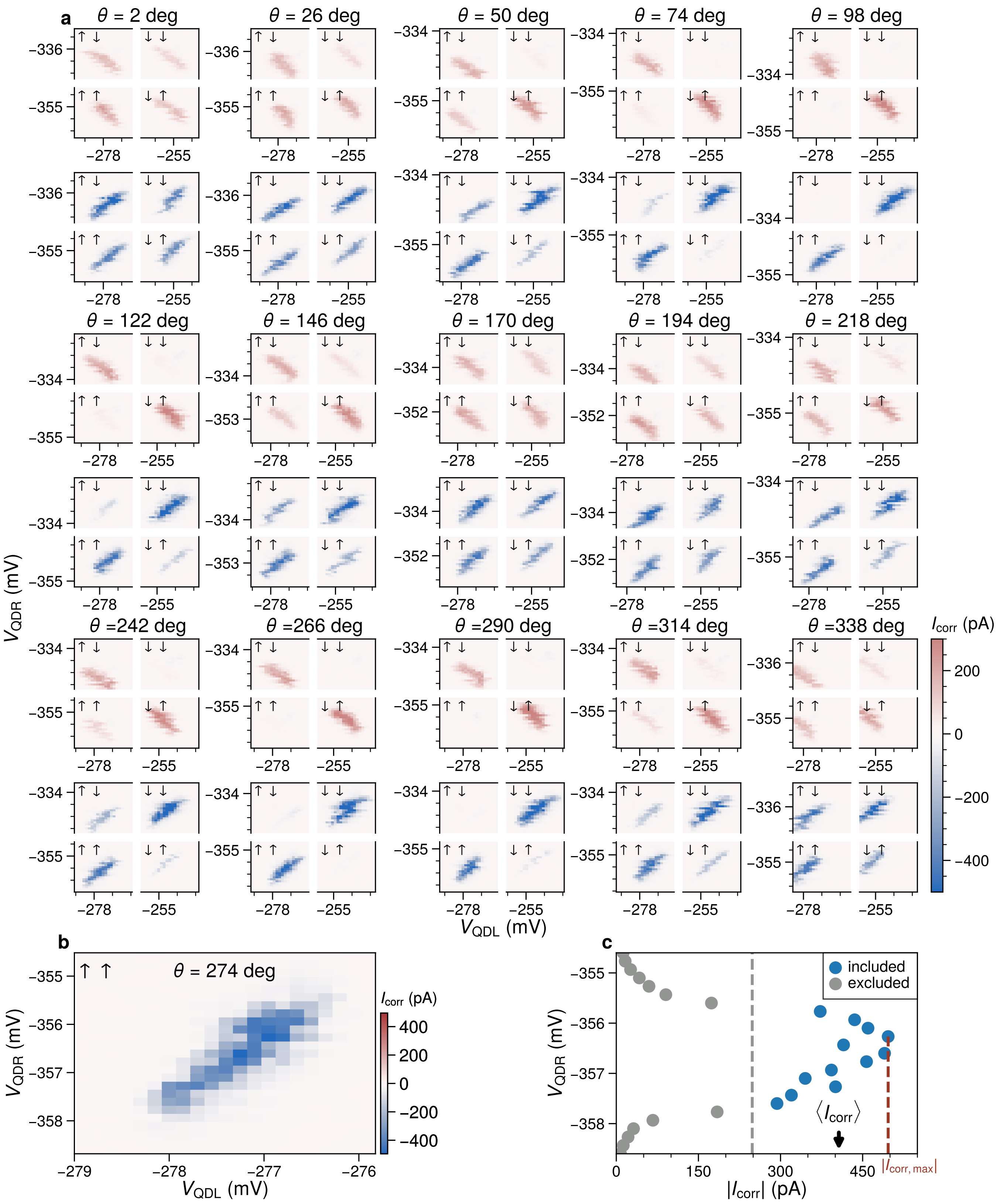

FIG. S7. **Raw data from Fig 4f and data extraction.** **(a)** Selection of raw data used for extracting the field angle dependence of CAR and ECT rates presented in Fig. 4f. For each angle the four quadrants with different spin configurations of I_{corr} are plotted for CAR (top panels) and ECT (bottom panels). A single quadrant is converted to a single data point to quantify the spin-selective rate of CAR and ECT at a specific angle of the magnetic field. The data extraction process is detailed in panel **(c)** for the measurement of ↑↑-ECT at $\theta = 274^{\circ}$ (plotted again in **(b)**). The maximum value of $|I_{\mathrm{corr}}|$ for each horizontal line-cut is extracted and plotted as a function of V_{QDR}. To exclude data at the edge of the bias window, a threshold is set at half of the maximally recorded $|I_{\mathrm{corr,max}}|$. The included values are labeled in blue, from which the average $\langle I_{\mathrm{corr}} \rangle$ and standard deviation is derived.

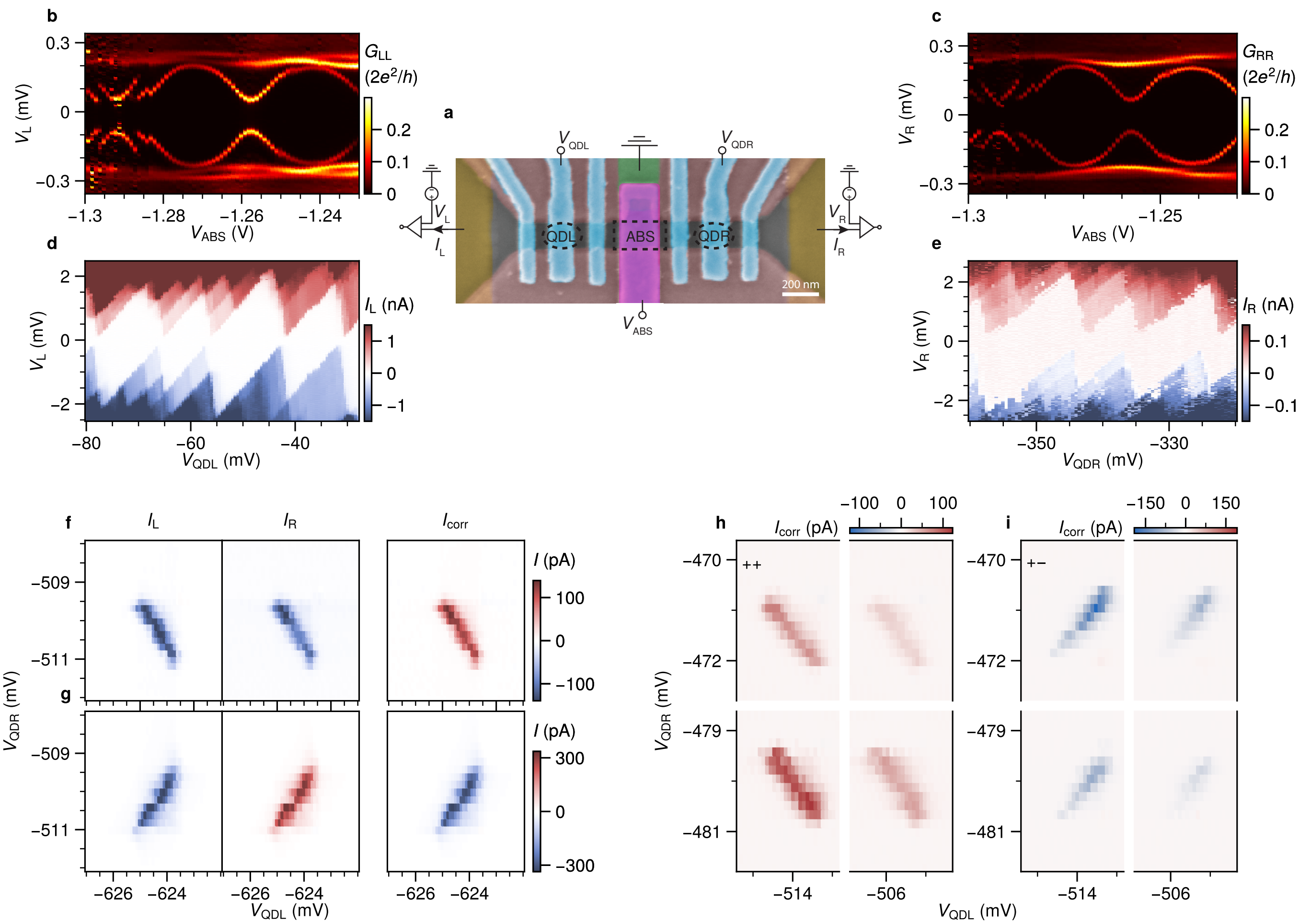

FIG. S8. **Measurements on additional device (Device 2).** **(a)** False-color scanning electron micrograph of Device 2, with the circuit diagram used for three-terminal measurements. Scale bar is 200 nm. The distance between the centers of the QDs is about 800 nm (in contrast to 600 nm for Device 1 presented in the main text). Tunneling spectroscopy measurements of the **(b)** local conductance G_{LL} and **(c)** G_{RR} as a function of V_{ABS} show correlated states, indicating the presence of an extended ABSs. Measured Coulomb diamonds for **(d)** QDL and **(e)** QDR. The extracted charging energy is about 1.2 meV for both dots. **(f)**,**(g)** Similar to Fig. 2, measured left current I_L, right current I_R and the calculated correlated current I_{corr}, for CAR ($V_L = V_R = 100\,\mu V$) and ECT ($V_L = 100\,\mu V$ and $V_R = -100\,\mu V$). Measurements over successive charge transitions reveal the expected spin blockade for **(h)** CAR (+,+) and **(i)** ECT (+,−) at zero magnetic field.
